# Massive Small Bowel Infaction: A Comparison of Two Cases

**DOI:** 10.4021/gr2009.07.1302

**Published:** 2009-07-20

**Authors:** James B Haddow, Ilyas Arshad, Andrew Redfern, Pradeep K Agarwal

**Affiliations:** a Colorectal Unit, Pilgrim Hospital, Boston, Lincolnshire, UK

**Keywords:** Small intestine, Infarction, Mesentery, Volvulus

## Abstract

We present two similar cases of massive small bowel infarction in which two different surgical approaches were employed, illustrating the advantages of the staged approach of damage control surgery. This comprises an initial operation, limited to bowel resection and temporary closure of the bowel ends, and a second performed after 48 to 72 hours where the bowel continuity is re-established. We strongly advocate such an approach as it appears to offer a quicker recovery with fewer complications.

## Introduction

Massive small bowel infarction is a life-threatening condition that requires immediate surgical intervention. We present two similar cases in which two different surgical approaches were employed, illustrating the advantages of the staged approach of damage control surgery.

## Case Report

### Case one

A 68 years old man was admitted with small bowel obstruction which was confirmed by a gastrograffin small bowel follow-through. He was treated expectantly, but over the next four days his condition deteriorated.

An emergency laparotomy revealed infarcted small bowel strangulated by internal herniation through adhesive bands. One third of the small bowel was resected and anastomosed using a stapling device.

Unfortunately four days later he developed peritonitis and a second laparotomy revealed further infarcted small bowel distal to the anastomosis. This required further resection and fashioning of an end jejunostomy and a caecostomy. He had a long post-operative recovery, requiring total parenteral nutrition for three months as an inpatient. This was complicated by recurrent septicaemia and in addition, he suffered constant excoriation of the skin around the stoma site. Four months after discharge, he underwent an elective laparotomy to restore the continuity of the bowel.

### Case two

A 60 years old woman presented with an insidious onset of epigastric pain associated with nausea and one episode of vomiting. She had previously undergone an abdominal hysterectomy.

Three days later she suddenly deteriorated. An emergency laparotomy revealed a small bowel volvulus that had twisted 780 degrees clockwise around the mesenteric root. Three metres of infarcted small bowel were resected and a stapling device was used to close the proximal and distal stumps separately. The abdomen was then washed out and closed without any attempt at anastomosis or stoma formation. This allowed swift transfer of the patient to the intensive care unit where she was stabilised with fluid replacement, haemofiltration, ionotropes, antibiotics and continued ventilation.

A planned re-look laparotomy was performed after 48 hours, where a further two centimetres of ischaemic small bowel were resected from the distal stump and continuity of the bowel was restored by anastomosing the remaining jejunum to the caecum with a stapling device. The patient then made a successful recovery and was discharged after 14 days.

## Discussion

Massive small bowel infarction is rare and has a high mortality rate. Data for small bowel infarction is scarce, but the overall mortality for acute mesenteric ischaemia is around 70 per cent [1].

Our two cases of small bowel infarction were similar in nature but were managed in two different ways. In the first, all necessary manoeuvres were performed in one operation: small bowel resection and primary anastomosis. However, the patient developed further ischaemia distal to the anastomosis. As a consequence there was considerable long-term morbidity.

In the second case, anastomosis was not attempted initially. A second laparotomy was performed after 48 hours, which allowed a further short segment of ischaemic bowel to disclose itself and thus permitted a confident anastomosis to be performed. The result was an uneventful recovery.

This staged approach, termed damage control surgery (DCS), has been developed in the trauma population. The rationale behind this approach lies in the physiology of an acutely traumatized patient who displays the lethal triad of hypothermia, acidosis and coagulopathy [2]. The interventions of definitive surgery, whilst repairing the injuries, actually contribute to the deranged physiology and delay full intensive care support. The abbreviated initial laparotomy of DCS is aimed at breaking this lethal triad and thus allowing the patient to be quickly stabilised in an intensive care setting. Definitive repairs can later be made more carefully under physiologically stable conditions.

Pringle first described the concept of DCS in 1908 in the treatment of hepatic injuries. The technique was popularised by Stone et al. in 1983, describing packing methods for bleeding and temporary manoeuvres for intestinal and urological injuries. The approach has since been widely adopted and in a review of 1001 patients who underwent DCS, the overall mortality and morbidity was 50 per cent and 40 per cent respectively [3]

More recently DCS has been adopted in non-trauma patients. Finlay et al. used DCS in the treatment of critically ill, general surgical patients and demonstrated a lower mortality rate than that predicted by the physiologic and operative severity score for the enumeration of mortality and morbidity (POSSUM) or Portsmouth-POSSUM: 7.1 per cent versus 64.5 per cent and 49.6 per cent respectively [4]. A group in Australia also included the use of diagnostic or therapeutic angiography in between the two operations to improve the outcome of patients with acute mesenteric ischaemia [5].

In conclusion, Patients who develop massive small bowel infarction are critically ill and it is important to consider their physiological state when operative measures are taken. A lengthy operation may worsen their condition and delay commencement of intensive care. The extent of the small bowel infarction is often not apparent at the time of the first operation. We strongly advocate the staged approach of damage control surgery in the management of small bowel infarction whereby the initial operation is limited to bowel resection and temporary closure of the bowel ends. A second laparotomy should be performed after 48 to 72 hours where the bowel continuity is re-established. Such an approach appears to offer a quicker recovery with fewer complications.
